# The Preventive Effect on Ethanol-Induced Gastric Lesions of the Medicinal Plant* Plumeria rubra*: Involvement of the Latex Proteins in the NO/cGMP/K_**ATP**_ Signaling Pathway

**DOI:** 10.1155/2015/706782

**Published:** 2015-12-14

**Authors:** Nylane Maria Nunes de Alencar, Rachel Sindeaux Paiva Pinheiro, Ingrid Samantha Tavares de Figueiredo, Patrícia Bastos Luz, Lyara Barbosa Nogueira Freitas, Tamiris de Fátima Goebel de Souza, Luana David do Carmo, Larisse Mota Marques, Marcio Viana Ramos

**Affiliations:** ^1^Departamento de Fisiologia e Farmacologia, UFC, Coronel Nunes de Melo 1127, Rodolfo Teófilo, 60430-270 Fortaleza, CE, Brazil; ^2^Centro Universitário Estácio do Ceará, Via Corpvs, Rua Eliseu Uchoa Becco, No. 600, Bairro Água Fria, 60810-270 Fortaleza, CE, Brazil; ^3^Departamento de Bioquímica e Biologia Molecular, UFC, Campus do Pici, Caixa Postal 6033, 60451-970 Fortaleza, CE, Brazil

## Abstract

*Plumeria rubra* (Apocynaceae) is frequently used in folk medicine for the treatment of gastrointestinal disorders, hepatitis, and tracheitis, among other infirmities. The aim of this study was to investigate the gastroprotective potential of a protein fraction isolated from the latex of* Plumeria rubra* (PrLP) against ethanol-induced gastric lesions and describe the underlying mechanisms. In a dose-dependent manner, the pretreatment with PrLP prevented ethanol-induced gastric lesions in mice after single intravenous administration. The gastroprotective mechanism of PrLP was associated with the involvement of prostaglandins and balance of oxidant/antioxidant factors. Secondarily, the NO/cGMP/K_ATP_ pathway and activation of capsaicin-sensitive primary afferents were also demonstrated as part of the mechanism. This study shows that proteins extracted from the latex of* P*.* rubra* prevent gastric lesions induced in experimental animals. Also, the results support the use of the plant in folk medicine.

## 1. Introduction

Peptic ulcer is a multifactorial disease that affects an increasing number of people worldwide. Etiological factors include emotional stress, improper diet, excessive ethanol ingestion, genetic factors, continuous or indiscriminate use of NSAIDs, and infection by* Helicobacter pylori* [1, 2]. Antagonists of histamine H2–6 receptors and inhibitors of the proton pump are currently the main classes of drugs used in the clinic for the treatment of peptic ulcer [3]. However, some adverse effects are associated with their long-term use, such as hypergastrinemia and an increased risk of* Helicobacter pylori* and* Clostridium difficile* infections [4, 5]. Furthermore, the high costs of these drugs are still concerns to be addressed. These inconsistencies stimulate the search for alternative or complementary strategies to improve the prevention and healing of ulcers.

Latex fluids have been reported to display numerous pharmacological properties. This is in good agreement with the traditional and folk medicinal use of latex-bearing plants worldwide [6, 7].* Plumeria rubra* is a laticifer plant, commonly known as the frangipani or temple tree and distributed mainly in tropical and subtropical regions [8]. It is used in folk medicinal purposes to treat or relieve fever, cold, cutaneous infections, tracheitis, gastrointestinal disorders, ureterolithiasis, and hepatitis and to induce coughing up. The traditional uses have been further certified by scientific documentation [9, 10]. Active phytoconstituents (plumericin and isoplumericin) isolated from* P*.* rubra* showed antialgal, antifungal, antibacterial, and molluscicidal effects and cytotoxic properties [11–13]. The n-hexane fraction of crude methanolic extract stem bark showed* in vitro* antimicrobial activity [14]. The flavone glycoside isolated from the flowers exhibited a significant reduction in serum triglycerides of animals [15]. More recently, the involvement of proteins in pharmacological activities of* P*.* rubra* was reported. A protease Plumerin-R was isolated from the latex by acetone precipitation and displayed anti-inflammatory activity [16]. In our previous findings, the soluble proteins extracted from the latex (PrLP) showed antioxidant and proteolytic activity [17] and endothelial relaxation of rat thoracic aortic rings [18]. Furthermore, studies with the proteins extracted from latex of other plants have suggested that the proteins are strongly associated with the pharmacological properties claimed by the popular medicine [6, 7]. In the present study, the aqueous protein fraction extracted from the latex of* P*.* rubra* was examined* in vivo* to determine its gastroprotective potential.

## 2. Materials and Methods

### 2.1. Latex Extraction and Protein Recovered

The fresh latex from* Plumeria rubra* L. (Jasmine) was collected in specimens growing in the Garden of Medicinal Plants, Universidade Federal do Ceará (UFC), Brazil. The plant material was identified by a taxonomist and a voucher specimen (number 15018) was deposited at Prisco Bezerra Herbarium of the UFC. Briefly, terminal branches were used to extract fresh latex. The fluid was taken into plastic tubes containing distilled water to yield a dilution ratio of 1 : 2 (v/v). The samples were centrifuged at 5,000 ×g at 10°C for 10 minutes. The precipitated rubber-like material was discarded and the supernatant was submitted to dialysis against distilled water using a membrane with a cutoff of 8000 Da. After 48 h, the nondialyzable material (PrLP) was obtained after centrifugation under the same conditions described above. The supernatant lyophilized was stored at 25°C until use. Soluble proteins from the latex of* Plumeria rubra* (PrLP) used in this study were previously characterized [17]. The summary of their properties will be presented later in this paper.

### 2.2. Animals

Adult male Swiss mice weighing 25 ± 3.0 g were obtained from the Central Animal House of the Universidade Federal do Ceará, Brazil. The animals were kept in plastic cages under controlled environmental conditions (12/12 h light/dark cycles, temperature 25°C, and humidity 55 ± 10%) with free access to water and fed with a commercial feed (Purina, Paulínia, SP, Brazil) until 16 hours before the experiments. All experimental procedures were handled according to the current* Guide for the Care and Use of Laboratory Animals* of the National Research Council after approval by the “Ethical Committee for Animal Use” of the Universidade Federal do Ceará (protocol number 57/2010).

### 2.3. Chemicals

Capsaicin, capsazepine, indomethacin, glibenclamide, diazoxide, L-arginine, N*ω*-nitro-L-arginine methyl ester (L-NAME), 1H-[1,2,4]oxadiazolo[4,3-a]quinoxalin-1-one (ODQ), and absolute ethanol were purchased from Sigma-Aldrich (St. Louis, MO, USA). Prostaglandin analog 16,16-dimethyl PGE2 (misoprostol) was purchased from Continental Pharma (Cytotec, Italy). N-Acetylcysteine was purchased from União Química (São Paulo, Brazil). All other chemicals were of analytical grade unless otherwise specified.

### 2.4. Ethanol-Induced Gastric Lesions

The animals were randomly distributed into six groups (*n* = 8 per group) and pretreated intravenously (i.v.) with vehicle (0.9% NaCl, 10 mL/kg; positive control group) or PrLP (0.05, 0.5, 5, or 50 mg/kg) or orally (p.o.) with N-acetylcysteine, the standard antioxidant drug (NAC, 750 mg/kg). After 30 minutes, a single intragastric dose of absolute ethanol (0.2 mL) was administered to positive control (vehicle) or PrLP groups and after 60 minutes to NAC group. One hour after ethanol treatments, the animals were sacrificed with anesthetic overdose. The stomachs were excised, opened along the greater curvature, and rinsed with saline (0.9%), according to the method described by Robert et al. [19]. The stomachs were scanned and the extension of the ulcerated area (%) was estimated using a computer planimetry program (ImageJ; National Institutes of Health, USA) [20]. After the statistical analysis of the data, the lower effective dose of the 4 doses tested was used for all other assays. Subsequently, gastric corpus samples were then weighed, frozen, and stored at −70°C until being assayed to determine glutathione (GSH) levels [21].

### 2.5. Microscopic Analyses

Samples of the gastric mucosa were fixed in formaldehyde (10%, v/v) and prepared in 0.01 M PBS, pH 7.2, over 24 h for histopathology procedures. Sections (5 *μ*m) were stained with hematoxylin-eosin to evaluate gastric mucosal injury according to the criteria of Laine and Weinstein [22], as follows: edema in the upper mucosa (0–4), hemorrhagic damage (0–4), epithelial cell loss (0–3), and the presence of inflammatory cells (0–3). Photomicrographs of sections were obtained with a Leica DM microscope equipped with a Leica DFC 280 camera (200x magnification).

### 2.6. Role of Prostaglandins in Gastroprotective Effect of the PrLP

The influence of endogenous prostaglandins was investigated as previously described by Morais et al. [23]. The animals (*n* = 8 per group) were pretreated with the cyclooxygenase inhibitor (indomethacin 10 mg/kg, p.o.), 60 min before the PrLP (0.5 mg/kg, i.v.) or a synthetic prostaglandin E1 analog (misoprostol 50 *μ*g/kg, p.o.). Other groups of animals were treated only with vehicle (0.9% NaCl, 10 mL/kg, i.v.), PrLP (0.5 mg/kg, i.v.), or misoprostol (50 *μ*g/kg, p.o.). After 30 minutes, a single intragastric dose of absolute ethanol (0.2 mL) was administered to positive control (vehicle) or PrLP groups and after 60 minutes to misoprostol groups. One hour after ethanol treatments, the animals were sacrificed with anesthetic overdose. The stomachs were excised and opened along the greater curvature [19]. The extension of the ulcerated area (%) was estimated using ImageJ software [20].

### 2.7. Role of Nitric Oxide in Gastroprotective Effect of the PrLP

The involvement of nitric oxide was investigated, as previously described [23]. The animals (*n* = 8 per group) were pretreated with inhibitor of the nitric oxide synthase (L-NAME 20 mg/kg) by intraperitoneal (i.p.) administration, 30 min prior to the treatment with PrLP (0.5 mg/kg, i.v.) or a substrate for nitric oxide synthase (L-arginine 600 mg/kg, i.p.). Other groups of animals were treated only with vehicle (0.9% NaCl, 10 mL/kg, i.v.), PrLP (0.5 mg/kg, i.v.), or L-arginine (600 mg/kg, i.p.). After 30 minutes, all animals received a single intragastric dose of absolute ethanol (0.2 mL). One hour later, the animals were sacrificed with anesthetic overdose. The stomachs were excised and opened along the greater curvature [19]. The extension of the ulcerated area (%) was estimated using ImageJ software [20]. Subsequently, samples from gastric mucosa were then weighed, frozen, and stored at −70°C until being assayed for NO_3_/NO_2_ production [24].

### 2.8. Evaluation of Transient Receptor Potential Vanilloid Type 1 (TRPV1) Triggers

The activation of capsaicin-sensitive primary afferents was investigated using a TRPV1 antagonist, capsazepine [25]. The animals (*n* = 8 per group) were pretreated with capsazepine (5 mg/kg, i.p.), 30 min prior to the treatment with PrLP (0.5 mg/kg, i.v.) or a vanilloid agonist (capsaicin 0.3 mg/kg, p.o.). Other groups of animals were treated only with vehicle (0.9% NaCl, 10 mL/kg, i.v.), PrLP (0.5 mg/kg, i.v.), or capsaicin (0.3 mg/kg, p.o.). After 30 minutes, a single intragastric dose of absolute ethanol (0.2 mL) was administered to positive control (vehicle) or PrLP groups and after 60 minutes to capsaicin groups. One hour after ethanol treatments, the animals were sacrificed with anesthetic overdose. The stomachs were excised and opened along the greater curvature [19]. The extension of the ulcerated area (%) was estimated using ImageJ software [20].

### 2.9. Evaluation of Soluble Guanylate Cyclase Activation

A soluble guanylate cyclase inhibitor (ODQ) was used to investigate the involvement of cGMP [26]. The animals (*n* = 8 per group) were pretreated with ODQ (10 mg/kg, i.p.), 30 min prior to the treatment with PrLP (0.5 mg/kg, i.v.). Other groups of animals were treated only with vehicle (0.9% NaCl, 10 mL/kg, i.v.) or PrLP (0.5 mg/kg, i.v.). After 30 minutes, all animals received a single intragastric dose of absolute ethanol (0.2 mL). One hour later, the animals were sacrificed with anesthetic overdose. The stomachs were excised and opened along the greater curvature [19]. The extension of the ulcerated area (%) was estimated using ImageJ software [20].

### 2.10. Role of ATP-Sensitive Potassium Channels (K_ATP_) in Gastroprotective Effect of PrLP

Pharmacological modulation of K_ATP_ with diazoxide (agonist) or glibenclamide (antagonist) was used to investigate the involvement of these channels in the gastroprotective effect of PrLP [22]. The animals (*n* = 8 per group) were pretreated with glibenclamide (5 mg/kg, i.p.), 30 min prior to the treatment with PrLP (0.5 mg/kg, i.v.) or diazoxide (3 mg/kg, i.p.). Other groups of animals were treated only with vehicle (0.9% NaCl, 10 mL/kg, i.v.), PrLP (0.5 mg/kg, i.v.), or diazoxide (3 mg/kg, i.p.). After 30 minutes, all animals received a single intragastric dose of absolute ethanol (0.2 mL). One hour later, the animals were sacrificed with anesthetic overdose. The stomachs were excised and opened along the greater curvature [19]. The extension of the ulcerated area (%) was estimated using ImageJ software [20].

### 2.11. Measurement of Nitrate/Nitrite Levels in the Gastric Mucosa

Nitrite levels in biopsy lysates from the gastric mucosa were determined indirectly as the total content of nitrite and nitrate (NO_3_^−^/NO_2_^−^) by a spectrophotometric method based on the Griess reaction [24]. Samples of the gastric mucosa were homogenized in 50 mM potassium phosphate buffer (pH 7.8) and centrifuged at 11,000 ×g for 15 minutes at 4°C. An aliquot of each sample (80 *μ*L) was incubated in a microplate with nitrate reductase for 12 h to convert NO_3_ into NO_2_. At room temperature (25°C), 100 *μ*L of Griess reagent (1% sulphanilamide in 1% phosphoric acid and 0.1% naphthalene diamine dihydrochloride in water) was added and incubated for 10 minutes. The optical densities were measured at 540 nm in a microplate reader. Nitrite concentrations in the samples were determined from a standard curve generated by different concentrations of sodium nitrite (0.1–100 mM). The data are expressed as micromoles of nitrite. All analyses were performed in triplicate and were reproduced without significant differences.

### 2.12. Glutathione (GSH) Levels in the Gastric Mucosa

Samples of the gastric mucosa were homogenized in a solution that contained 1 mL of a 0.02 M EDTA cooled solution, 320 *μ*L of distilled water, and 400 *μ*L of trichloroacetic acid (TCA) 50% (w/v). The homogenates were centrifuged at 3000 ×g for 15 minutes. The supernatants (400 *μ*L) were mixed with 800 *μ*L of Tris buffer (40 mM, pH 8.9) and 5,5′-dithiobis(2-nitrobenzoic acid) (DTNB, 10 mM) was added. The absorbance was measured within 3 minutes after addition of DTNB at 412 nm against a blank reagent without homogenate [21]. The absorbance values were extrapolated from a reduced glutathione standard curve and expressed as NP-SH/g of stomach tissue.

### 2.13. Statistical Analysis

Values are expressed as the mean ± standard errors mean (SEM) or median. Analysis of Variance (ANOVA) followed by Student-Newman-Keuls test was used to compare means and Kruskal-Wallis nonparametric test, followed by Dunn's test, to compare medians; *P* < 0.05 was defined as statistically significant.

## 3. Results and Discussion

PrLP comprises the water soluble protein fraction extracted from the whole latex of* Plumeria rubra*. The antioxidative enzymes ascorbate peroxidase and superoxide dismutase were detected in PrLP while catalase was absent. Chitinases were also found. Proteolytic enzymes that were best inhibited by E-64 were reported [17]. These authors reported that PrLP represents nearly 0.33 mg of protein in 1 mL of crude latex. PrLP is free of other metabolites, mainly those produced by the secondary metabolism. Water insoluble compounds are lost by precipitation along dialyses in water and the water soluble ones are lost through the dialyses membrane. Chemical assays for measurement of saponins, flavonoids, phenols, tannins, triterpenes, and alkaloids on PrLP have failed (data not shown). At least the overall profile of proteins present in PrLP can be found in the studies of de Freitas et al. [17] cited above.

Experimental studies to highlight gastroprotective substances have been performed using a model of ethanol-induced gastric lesions [27–29]. Here, we investigated the gastroprotective potential of PrLP, since the properties of latex proteins in different pharmacological models have been successfully confirmed. The venous route for sample administration was selected as the first approach in order to avoid the physiochemical instability and enzymatic barrier of proteins. Moreover, the influence of enterohepatic recirculation could reduce the bioavailability of the soluble proteins. We have initiated approaches to determine the potential subchronic toxicity of PrLP in animals. Serum level of urea and the enzymatic activities of alanine aminotransferase (ALT or TGP) and aspartate aminotransferase (AST or TGO) determined with standardized diagnostic kits were normal in animals given PrLP at tested doses (unpublished data).

**Figure 1:**
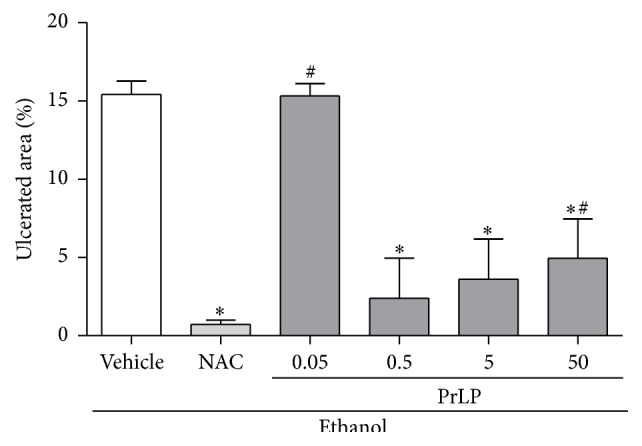
Ethanol-induced gastric lesions. Animals were sacrificed, and their stomachs were immediately excised to evaluate the extension of the ulcerated area. Data are expressed as the mean ± standard error mean (SEM) of the number of lesions (8 animals per group), expressed in percent. ^*∗*^ *P* < 0.05 indicates a significant difference compared with the positive control group (vehicle); ^#^ *P* < 0.05 indicates a significant difference compared with animals treated with NAC (ANOVA, Newman-Keuls test).

In a dose-dependent manner, the pretreatment with PrLP prevented the appearance of gastric mucosal ulceration when compared with positive control group (vehicle) (*P* < 0.05). The effect was similar to that observed in the N-acetylcysteine (NAC) group. The highest gastroprotective effect of PrLP occurred at a dose of 0.5 mg/kg (Figure 1).

**Figure 2:**
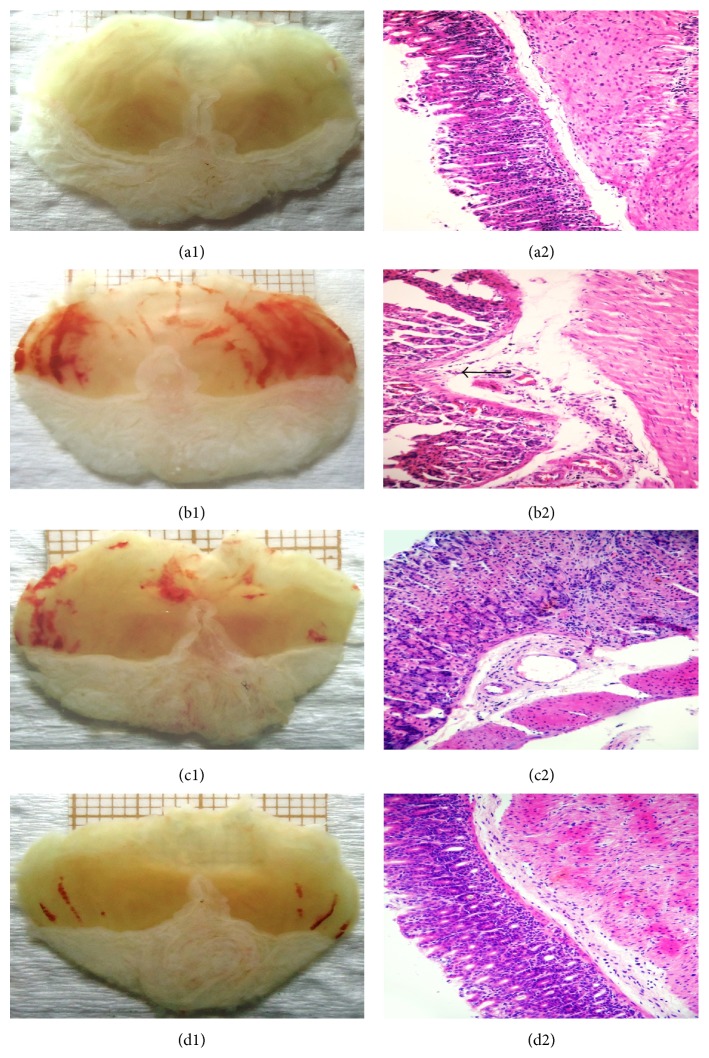
Gastric mucosal lesions: macro- and microscopic aspects. Animals were sacrificed, and their stomachs were opened along the greater curvature. For macroscopic evaluation, images were selected from animals belonging to the corresponding experimental groups (a1–d1). Samples of the gastric mucosa were removed to perform histological analyses (a2–d2). Hematoxylin-eosin stained sections were employed to obtain photomicrographs and to estimate hemorrhage, loss of epithelial cells, and inflammatory infiltrates (arrow) among the different experimental groups: (a1, a2) negative control group, (b1, b2) positive control group, (c1, c2) PrLP 0.5 mg/kg, i.v., and (d1, d2) NAC (200x magnification).

Macro- and microscopic aspects of the gastric mucosa are shown in Figures 2(a)–2(d) and Table 1. No type of lesion was observed in the samples of stomach from the negative control group (saline), as seen by the gastric epithelia integrity (Figures 2(a1) and 2(a2)). Animals pretreated with vehicle (positive control group) and subjected to intragastric absolute ethanol presented a significant number of lesions on the gastric mucosa, with intense signs of hemorrhage and loss of epithelial cells when compared with negative control group (Figures 2(b1) and 2(b2)). The groups of animals pretreated with PrLP or NAC were protected from alterations induced by ethanol (Figures 2(c1), 2(c2), 2(d1), and 2(d2)).

The genesis of ethanol-induced gastric lesions is multifactorial and is associated with a decrease in the intrinsic gastric mucosal defense mechanisms or an increase in aggressive factors, mainly related to changes in the microcirculation and oxidative stress [26]. The existence of cytoprotective effect associated with a significant reduction of the ulcer index, besides free radical scavenging activity, was previously pointed to ethanol and chloroform extract of* Plumeria rubra* [30]. However, our data shown in this work suggest that the gastroprotective effect observed on animals submitted to induced gastric ulcer was mediated by the latex protein fraction instead of other metabolites.

**Figure 3:**
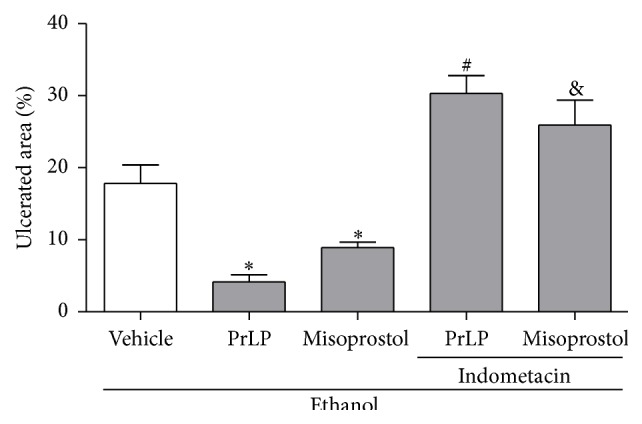
Involvement of prostaglandins in ethanol-induced gastric lesions. Animals were sacrificed, and their stomachs were immediately excised to evaluate the extension of the ulcerated area. Data are expressed as the mean ± standard error mean (SEM) of the number of lesions (8 animals per group), expressed in percent. ^*∗*^ *P* < 0.05 indicates a significant difference compared with the positive control group (vehicle); ^#^ *P* < 0.05 indicates a significant difference compared with animals treated only with PrLP; ^&^ *P* < 0.05 indicates a significant difference compared with animals treated only with misoprostol (ANOVA, Newman-Keuls test).

It is well known that prostaglandin E2 (PGE2), an eicosanoid, has gastroprotective effects through stimulating bicarbonate and mucus release, also provoking vasodilation and increased blood flow [31–33]. The effects of PGE2 on ethanol-induced gastric lesions are due to an increase in intracellular cGMP, mediated by an increase in intracellular calcium concentration and nitric oxide production [34, 35]. The involvement of prostaglandins in the gastroprotective mechanism of PrLP was investigated through pharmacological modulation with indomethacin (an inhibitor of prostaglandin synthesis). A significant extension of the gastric mucosal ulceration was observed in animals pretreated with vehicle (positive control group) and subjected to intragastric absolute ethanol (Figure 3). The appearance of injuries was significantly prevented by the pretreatment with PrLP or misoprostol, when compared with the positive control group (vehicle) (*P* < 0.05). However, the pretreatment of animals with indomethacin reversed the gastroprotective effects promoted by PrLP or misoprostol, significantly increasing the ethanol-induced gastric mucosal lesions (*P* < 0.05).

These results strongly indicate that PrLP acts through the involvement of prostaglandins, possibly stimulating the production of this mediator. Similarly, the protective response of chloroform and ethanolic extract of leaves from* P*.* rubra* against ethanol-induced gastric lesions was also a suggestion of its effect on prostaglandin synthesis [22].

**Figure 4:**
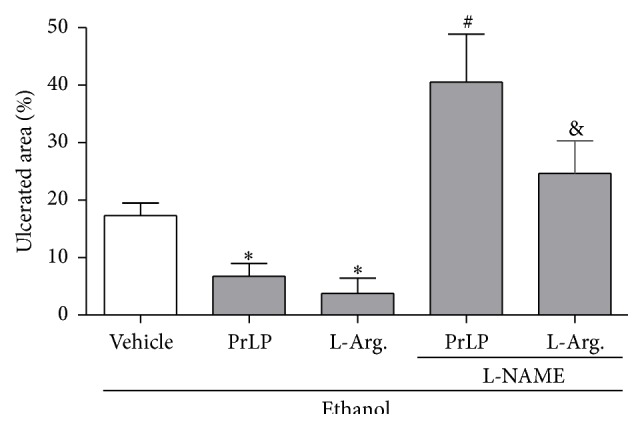
Involvement of nitric oxide in ethanol-induced gastric mucosal lesions. Animals were sacrificed, and their stomachs were immediately excised to evaluate the extension of the ulcerated area. Data are expressed as the mean ± standard error mean (SEM) of the number of lesions (8 animals per group), expressed in percent. ^*∗*^ *P* < 0.05 indicates a significant difference compared with the positive control group (vehicle); ^#^ *P* < 0.05 indicates a significant difference compared with animals treated only with PrLP; ^&^ *P* < 0.05 indicates a significant difference compared with animals treated only with L-arginine (ANOVA, Newman-Keuls test).

**Figure 5:**
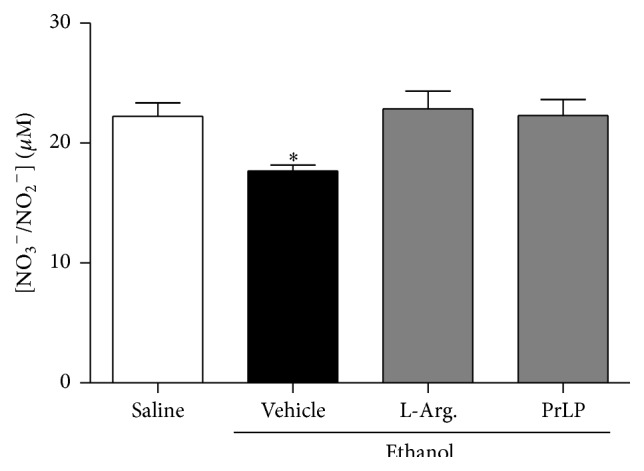
Nitrite levels in gastric mucosa. Animals were sacrificed, and their stomachs were immediately excised. Samples of the gastric mucosa were removed to determine nitrite levels by the Griess reaction. Data are the mean of three independent experiments and are expressed as the mean ± standard error mean (SEM) of nitric oxide (NO_3_/NO_2_) levels (*μ*M). ^*∗*^ *P* < 0.05 indicates a significant difference compared with the negative control group (saline) (ANOVA, Newman-Keuls test).

Gastric mucosal defense is also mediated physiologically by nitric oxide (NO) through blood flow regulation, mucus release, and inhibition of inflammatory infiltrates [36–38]. Thus, drugs that block the synthesis of NO exacerbate the lesions associated with ethanol. Therefore, an inhibitor of NO synthesis (L-NAME) was used in order to observe the involvement of NO in the gastroprotective effect of PrLP. A significant extension of the gastric mucosal ulceration was observed in animals pretreated with vehicle (positive control group) and subjected to intragastric absolute ethanol (Figure 4). The pretreatment with PrLP (i.v.) or L-arginine (i.p.) significantly prevented the appearance of lesions compared with the positive control group (*P* < 0.05). The gastroprotective effects promoted by PrLP or L-arginine were reversed in the group of animals pretreated with L-NAME (i.p.), which significantly increased ethanol-induced gastric lesions (*P* < 0.05). As demonstrated, nitric oxide was involved in the gastroprotective effects of PrLP. To confirm this hypothesis, stomach samples were used to measure nitrite/nitrate levels. Animals pretreated with vehicle (positive control group) and submitted to intragastric absolute ethanol exhibited reduced levels of nitrite in the gastric mucosa, when compared with the negative control group (*P* < 0.05) (Figure 5). The reduction in nitrite levels induced by ethanol was significantly prevented in the group of animals pretreated with PrLP or L-arginine (*P* < 0.05).

These results confirm the involvement of nitric oxide in the gastroprotective effect of PrLP, probably by constitutive overexpression of nitric oxide synthases (NOS) and their efficiency on catalytic actions. According to the published literature, nitric oxide increases vascular permeability and prostaglandin production in the gastric mucosa. Moreover, its effects on gastric microcirculation and the synthesis of mucus also result from cooperative activity with prostaglandins [39, 40].

**Figure 6:**
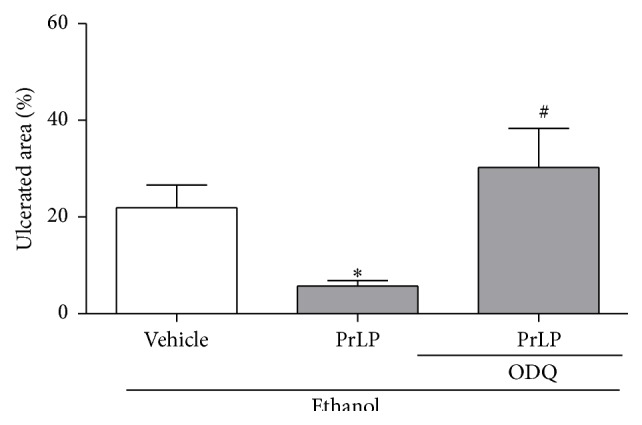
Involvement of the NO-sGS-cGMP signaling system in ethanol-induced gastric lesions. Animals were sacrificed, and their stomachs were immediately excised to evaluate the extension of the ulcerated area. Data are expressed as the mean ± standard error mean (SEM) of the number of lesions (8 animals per group), expressed in percent. ^*∗*^ *P* < 0.05 indicates a significant difference compared with the positive control group (vehicle); ^#^ *P* < 0.05 indicates a significant difference compared with animals treated only with PrLP (ANOVA, Newman-Keuls test).

It is well known that the vasodilator effect of NO is mediated by the stimulation of the enzyme soluble guanylyl cyclase (sGS) and the consequent release of cGMP [41]. In experimental models, a highly selective irreversible heme-site inhibitor of soluble guanylyl cyclase, known as ODQ (1H-[1,2,4]oxadiazolo[4,3-a]quinoxalin-1-one), has been used to identify the correlation of gastroprotective substances with the NO-sGS-cGMP signaling system [42, 43]. According to this approach, animals pretreated with vehicle (positive control group) and subjected to intragastric absolute ethanol exhibited a significant extension of the gastric mucosal ulceration (Figure 6). The appearance of lesions was significantly prevented in the group of animals pretreated with PrLP (i.v.). Another group of animals was pretreated with ODQ, in which the protective effects of PrLP against ethanol-induced gastric mucosal injury were reversed (*P* < 0.05). The same involvement of the NO-sGS-cGMP signaling system was also used to explain the gastroprotective mechanism of sildenafil (a drug commercially used to treat erectile dysfunction) by Medeiros et al. [42].

**Figure 7:**
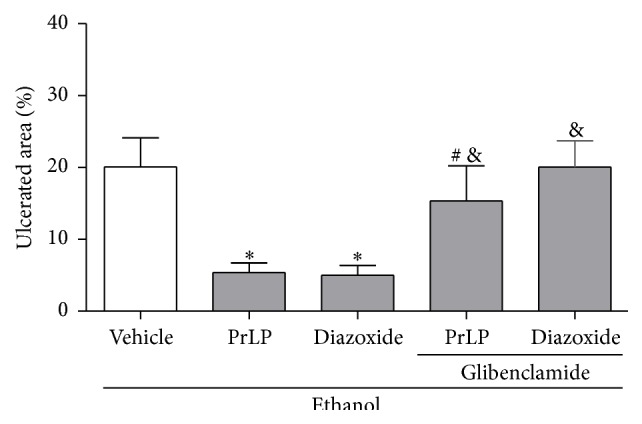
Involvement of ATP-dependent potassium channel (K_ATP_) in ethanol-induced gastric mucosal lesions. Animals were sacrificed, and their stomachs were immediately excised to evaluate the extension of the ulcerated area. Data are expressed as the mean ± standard error mean (SEM) of the number of lesions (8 animals per group), expressed in percent. ^*∗*^ *P* < 0.05 indicates a significant difference compared with the positive control group (vehicle); ^#^ *P* < 0.05 indicates a significant difference compared with animals treated only with PrLP; ^&^ *P* < 0.05 indicates a significant difference compared with animals treated only with diazoxide (ANOVA, Newman-Keuls test).

An ATP-dependent potassium channel (K_ATP_) is associated with the regulation of blood flow, acid secretion, and muscle contractility of the gastric mucosa [44]. Some compounds, such as diazoxide, inhibit ethanol-induced gastric mucosal damage through the opening of K_ATP_ channels, while glibenclamide, a blocker of these channels, attenuates gastric injuries [25, 45, 46]. Thus, to assess the contribution of K_ATP_ channels to the gastroprotective effects of PrLP, pharmacological approaches were employed. A significant extension of the gastric mucosal ulceration was observed in the group of animals pretreated with vehicle (positive control group) and subjected to intragastric absolute ethanol. The damage in the gastric mucosa was significantly prevented by the pretreatment with PrLP (i.v.) or diazoxide (i.p.) (*P* < 0.05). In another group of animals, the prior administration of glibenclamide (i.p.) reversed the gastroprotective effects promoted by PrLP or diazoxide against ethanol-induced gastric mucosal injury (*P* < 0.05) (Figure 7).

The gastroprotective mechanisms involving prostaglandins and nitric oxide are related to the activation of the guanylyl cyclase enzyme and release of the intracellular second messenger cyclic GMP (cGMP). Moreover, the activation of ATP-dependent potassium channels may occur in response to nitric oxide and cyclic GMP [26, 47, 48]. Therefore, this set of results suggests that the NO/cGMP/K_ATP_ pathway is of primary importance in the gastroprotective effect of PrLP.

**Figure 8:**
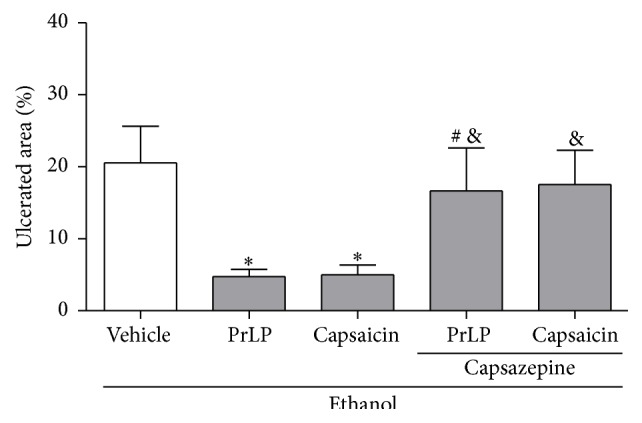
Involvement of transient receptor potential vanilloid 1 (TRPV1) in ethanol-induced gastric lesions. Animals were sacrificed, and their stomachs were immediately excised to evaluate the extension of the ulcerated area. Data are expressed as the mean ± standard error mean (SEM) of the number of lesions (8 animals per group), expressed in percent. ^*∗*^ *P* < 0.05 indicates a significant difference compared with the positive control group (vehicle); ^#^ *P* < 0.05 indicates a significant difference compared with animals treated only with PrLP; ^&^ *P* < 0.05 indicates a significant difference compared with animals treated only with capsaicin (ANOVA, Newman-Keuls test).

Capsaicin-sensitive sensory nerves are involved as a defense system to protect against gastric damage. The mechanism involves receptor stimulation at the plasma membrane, primarily of the transient receptor potential vanilloid type 1 (TRPV1) [49, 50]. A vanilloid antagonist capsazepine has been used to determine the involvement of these receptors as part of the gastroprotective mechanism for different substances [51, 52]. A significant extension of the gastric mucosal ulceration was observed in the group of animals pretreated with vehicle (positive control group) and subjected to intragastric absolute ethanol. As expected, PrLP (i.v.) and capsaicin (p.o.) significantly prevented the appearance of injuries when compared with the positive control group (*P* < 0.05). In the group of animals pretreated with vanilloid antagonist capsazepine, the gastroprotective effect of PrLP (i.v.) and capsaicin (p.o.) was significantly reduced (*P* < 0.05) (Figure 8). Our results indicate that the gastroprotective effect of PrLP is also mediated by the activation of capsaicin-sensitive primary afferents. A similar profile was observed in the gastroprotective effect of barbatusin and 3-beta-hydroxy-3-deoxibarbatusin, diterpenes able to stimulate receptor potential vanilloid type 1 (TRPV1) and promote an increase in nitric oxide, displaying mucosal defense through both of these mechanisms [22].

**Figure 9:**
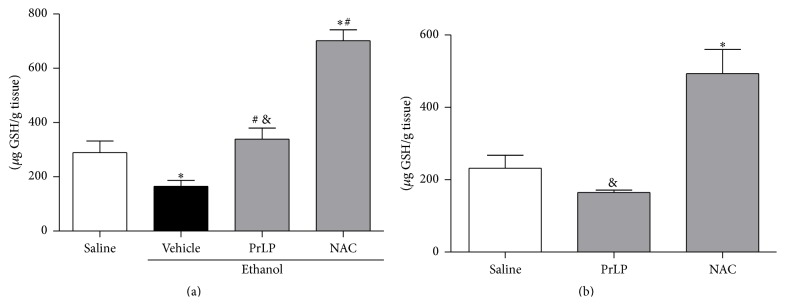
Glutathione (GSH) levels in ethanol-induced gastric lesions. Animals were sacrificed, and their stomachs were immediately excised. Samples of the gastric mucosa from animals subjected to ethanol (a) or not (b) were removed to determine glutathione levels. Data are the mean of three independent experiments and are expressed as the mean ± standard error mean (SEM) of *µ*g GSH/g of tissue. ^*∗*^ *P* < 0.05 indicates a significant difference compared with the negative control group (saline); ^#^ *P* < 0.05 indicates a significant difference compared with the positive control group (vehicle); ^&^ *P* < 0.05 indicates a significant difference compared with the NAC group (ANOVA, Newman-Keuls test).

Ethanol-induced gastric mucosal injury is related to the generation of free radicals and imbalance of oxidant/antioxidant factors [53, 54]. Clinical and experimental evidence suggest that antioxidant substances may promote gastroprotective effects [31, 52]. Therefore, grounded by studies that showed the antioxidative and proteolytic activities of laticifer cells of* P*.* rubra* [17], the involvement of PrLP in the antioxidant defense mechanisms was investigated through determination of glutathione (GSH) levels in the gastric mucosa. Animals from the negative control group (saline) displayed glutathione (GSH) levels within normal range, according to the published literature [55]. Animals pretreated with vehicle (positive control group) and subjected to intragastric absolute ethanol displayed significant mucosal GSH depletion when compared with the negative control group (*P* < 0.05). GSH levels were significantly (*P* < 0.05) restored in animals pretreated with NAC (p.o.) or PrLP (i.v.) before administration of intragastric absolute ethanol (Figure 9(a)). A second approach was taken to verify whether PrLP acts by increasing the levels of GSH without influencing ethanol-induced gastric mucosal injury. GSH levels were not altered in animals pretreated with PrLP when compared with the negative control group, while NAC promoted an increase, regardless of the deleterious effects promoted by ethanol (Figure 9(b)).

## 4. Conclusions

Soluble proteins from the latex of* Plumeria rubra* (PrLP) prevented ethanol-induced gastric lesions through the involvement of prostaglandins and balance of oxidant/antioxidant factors. Secondarily, the NO/cGMP/K_ATP_ pathway and activation of capsaicin-sensitive primary afferents were also demonstrated as part of the mechanism. This study also suggests that the use of the latex of* P*.* rubra* in folk medicine is pertinent. The diversity of manners in which people use latex as alternative medicine (as topical application or oral ingestion) encourages the scientific studies. However, it still represents a challenge to understand the true potentialities of latex compounds and determine the toxic potential and thus establish the best practices for their use.

## Figures and Tables

**Table 1:** Semi-quantitative evaluation of gastric lesions.

Groups	Microscopic scores		
	Hemorrhage	Loss epithelial cell	Inflammatory infiltrate
Negative control group	0 (0-0)	0 (0-0)	0 (0-0)
Positive control group	4 (4-4)^*∗*^	3 (2-3)^*∗*^	1 (0-1)
PrLP	0.5 (0-1)^#^	1 (1-1)^#^	0 (0-1)
NAC	0 (0-0)^#^	1 (1-1)^#^	0 (0-1)

Data represent the median and range of scores from two separate experiments: (0) absent, (1) mild, (2) moderate, (3) intense, (4) edema in the upper mucosa, hemorrhagic damage, epithelial cell loss and the presence of inflammatory cells. ^*∗*^ *P* < 0.05 indicates a significant difference compared with the negative control group. ^#^ *P* < 0.05 indicates a significant difference compared with the positive control group (vehicle). (*n* = 8 animals/group, Kruskal-Wallis test followed by Dunn's test).
